# Chronic Delivery of Antibody Fragments Using Immunoisolated Cell Implants as a Passive Vaccination Tool

**DOI:** 10.1371/journal.pone.0018268

**Published:** 2011-04-20

**Authors:** Osiris Marroquin Belaunzaran, Maria Isabel Cordero, Veronica Setola, Siro Bianchi, Carmela Galli, Nicolas Bouche, Vladimir Mlynarik, Rolf Gruetter, Carmen Sandi, Jean-Charles Bensadoun, Maurizio Molinari, Patrick Aebischer

**Affiliations:** 1 Neurodegenerative Studies Laboratory, Brain Mind Institute, Ecole Polytechnique Fédérale de Lausanne, Lausanne, Switzerland; 2 Laboratory of Behavioral Genetics, Brain Mind Institute, Ecole Polytechnique Fédérale de Lausanne, Lausanne, Switzerland; 3 Institute for Research in Biomedicine, Bellinzona, Switzerland; 4 Laboratory for Functional and Metabolic Imaging, Ecole Polytechnique Fédérale de Lausanne, Lausanne, Switzerland; University of Delhi, India

## Abstract

**Background:**

Monoclonal antibodies and antibody fragments are powerful biotherapeutics for various debilitating diseases. However, high production costs, functional limitations such as inadequate pharmacokinetics and tissue accessibility are the current principal disadvantages for broadening their use in clinic.

**Methodology and Principal Findings:**

We report a novel method for the long-term delivery of antibody fragments. We designed an allogenous immunoisolated implant consisting of polymer encapsulated myoblasts engineered to chronically release scFv antibodies targeted against the N-terminus of the Aβ peptide. Following a 6-month intracerebral therapy we observed a significant reduction of the production and aggregation of the Aβ peptide in the APP23 transgenic mouse model of Alzheimer's disease. In addition, functional assessment showed prevention of behavioral deficits related to anxiety and memory traits.

**Conclusions and Significance:**

The chronic local release of antibodies using immunoisolated polymer cell implants represents an alternative passive vaccination strategy in Alzheimer's disease. This novel technique could potentially benefit other diseases presently treated by local and systemic antibody administration.

## Introduction

Therapeutic monoclonal antibodies (mAbs) with more than 20 products in clinical use and over 200 candidates in clinical investigation constitute a promising avenue for the treatment of several major diseases including autoimmune, cardiovascular, infectious diseases, cancer and inflammation [1], [2], [3]. Furthermore, development of novel antibody targets for the treatment of several neurological diseases such as Alzheimer's disease (AD) is being currently investigated [4], [5], [6], [7], [8]. However, major drawbacks that presently limit the use of therapeutic antibodies following systemic delivery is related to the poor distribution at the targeted tissues, inadequate pharmacokinetics, and elevated costs of manufacture [9], [10].

The development of new methods for the continuous delivery of antibodies and/or its fragments that would allow reduction of interventions, prolonged retention at the targeted site, slow clearance and low cost of goods is therefore highly desirable.

In the present work, we propose a novel way to potentially release mAbs or antibody fragments in targeted tissues for extended periods of time using semipermeable polymeric cell implants. Surrounding genetically engineered cells producing mAbs and/or antibody fragments with a synthetic permselective membrane minimizes immunological responses by avoiding cell-to-cell contact between the host tissue and the encapsulated cells, while its design and porosity allows the inward diffusion of nutrients, oxygen and the outward diffusion of antibodies into the implanted tissue.

We show the feasibility of using an immunoisolated polymer implant loaded with genetically engineered C2C12 mouse myoblasts cells, to secrete single-chain fragment variable (scFv) antibodies. As proof-of-concept, we tested this technology as an immunotherapeutical approach for the treatment of AD using a transgenic mouse model of the disease. Implants releasing scFv antibodies placed in the brain parenchyma of APP23 transgenic mice proved to be capable of continuously process, express and secrete the scFvβ1 [11] antibody fragment targeted against the EFRH epitope of the Aβ peptide, the characteristic hallmark of AD brain pathology [12]. *In situ* chronic expression of scFvβ1 following a six-month immunotherapy in 14-months old APP23 mice reduced the accumulation and production of Aβ as analyzed with histological and biochemical markers. Functional assessment in mice showed significant behavioral recovery of anxiety and memory traits.

These results show that this novel technique to deliver antibodies into targeted tissues can serve as an alternative approach for the treatment of AD and potentially other major diseases treated by passive vaccination strategies.

## Results

### Reduction of Aβ *in vitro* following cell exposure to single chain and monoclonal antibodies against human APP

**Figure 1:**
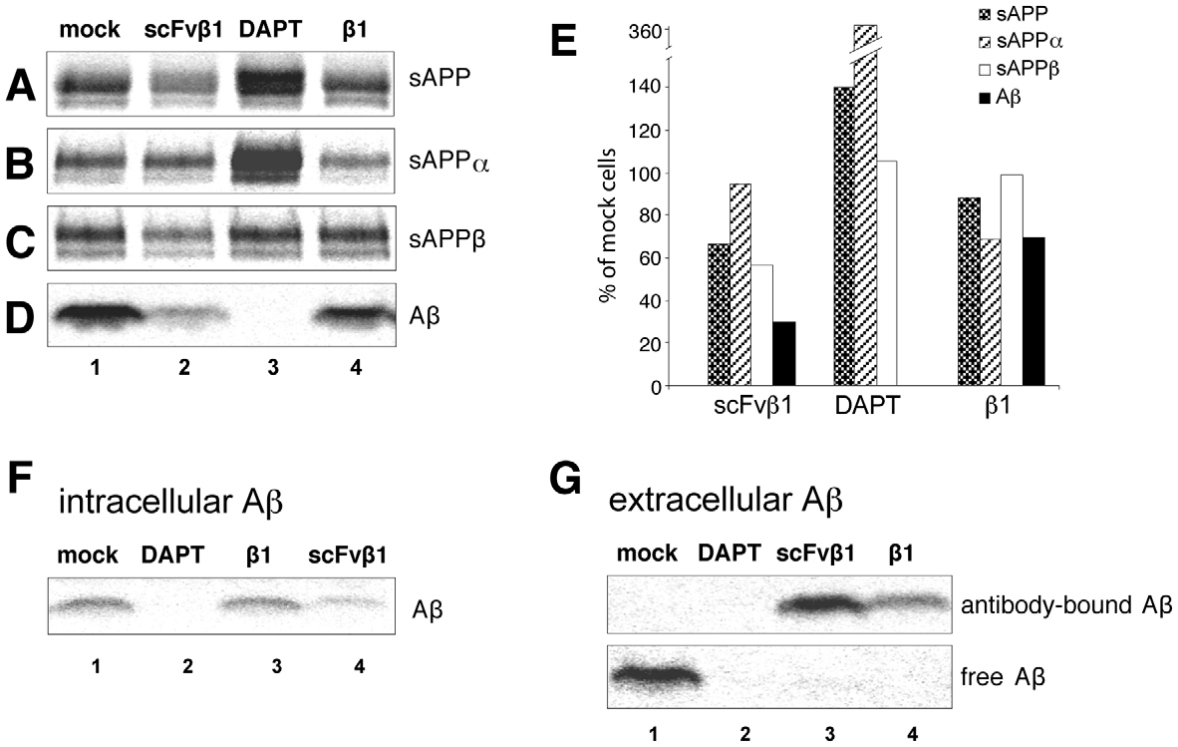
scFvβ1 decreases the accumulation and production of Aβ and sAPPβ by interfering with the BACE1 cleavage of huAPP. (**A–E**) Immunoprecipitations from cell protein extracts reveal that scFvβ1 added extracellularly to CHO mutant huAPP cells reduces the amount of soluble amyloid precursor protein beta (sAPPβ) by specifically interfering with the BACE1 cleavage site (**C**, **E**), thus decreasing the production of Aβ (**D**, **E**), and of the total soluble amyloid precursor protein (sAPP) (**A**, **E**). (**A–E**) The addition of β1 monoclonal antibody does not interfere with the BACE1 cleavage site of sAPPβ (A–E). (**A–E**) DAPT as positive control treatment abolishes the total production of Aβ by inhibiting the γ-secretase activity, favoring the alpha cleavage of APP. (**E**) All densitometry values of immunoprecipitations are plotted as the % change against mock cells. (**F**) Intracellular accumulation and production of Aβ is considerably reduced only in the presence of scFvβ1. (**G**) Aβ released from the cells in the culture medium was completely bound to either β1 or scFvβ1 antibody molecules.

scFvβ1 is a single chain antibody recognizing the EFRH tetrapeptide adjacent to the beta secretase cleavage site of human amyloid precursor protein (APP) [11]. We previously showed that intracellular expression of scFvβ1 resulted in association with newly synthesized APP in the endoplasmic reticulum. Formation of this complex shielded the beta secretase cleavage site of APP thus substantially reducing Aβ production [11]. Here we compared consequences on APP processing of extracellular administration of scFvβ1 or of the template β1 monoclonal antibody [13] to cultured CHO cells expressing the Swedish variant of human APP. When added to the culture medium the 27 kDa recombinant scFvβ1 bound to surface exposed APP and reduced the shedding of the APP ectodomain by 40% in a dose-dependent manner (**Figure 1A, E**). The α-secretase cleavage was poorly affected upon cell incubation with scFvβ1 (<10% of reduction, **Figure 1B, E**), while the β-secretase cleavage by BACE1 (β-site APP cleaving enzyme) was reduced by 50% compared to mock-treated cells (**Figure 1C, E**). Altogether, exposure to scFvβ1 substantially reduced intracellular production and accumulation of Aβ (**Figure 1D–G**).

Addition of the β1 monoclonal to the cell culture medium weakly affected shedding of the APP ectodomain (approximately 10% reduction, **Figure 1A, E**). This is consistent with an impaired access of the bulky, ∼180 kDa full-length antibody molecule to the EFRH epitope located close to the transmembrane domain of APP. Reduction of total Aβ (**Figure 1D, E**) and the fraction of Aβ trapped intracellularly (**Figure 1F**) was also significantly smaller compared to cells exposed to the scFvβ1. As control treatment we used DAPT, a potent inhibitor of γ-secretase [14] that abolishes the generation of Aβ (**Figure 1D–G**).

Despite the different capacity to actively interfere with Aβ production, both the single chain and the monoclonal antibodies efficiently buffered the Aβ released by cells in the culture medium. In fact, no free Aβ was detected in the supernatant after exposure to scFvβ1 or to β1, with all secreted Aβ being associated with the antibodies (**Figure 1G**). These data show that *in situ* delivery of scFvβ1 represents a powerful strategy with potential for beneficial interference with Aβ generation and for buffering neurotoxic Aβ forms.

### Design of a polymeric cell implant

**Figure 2:**
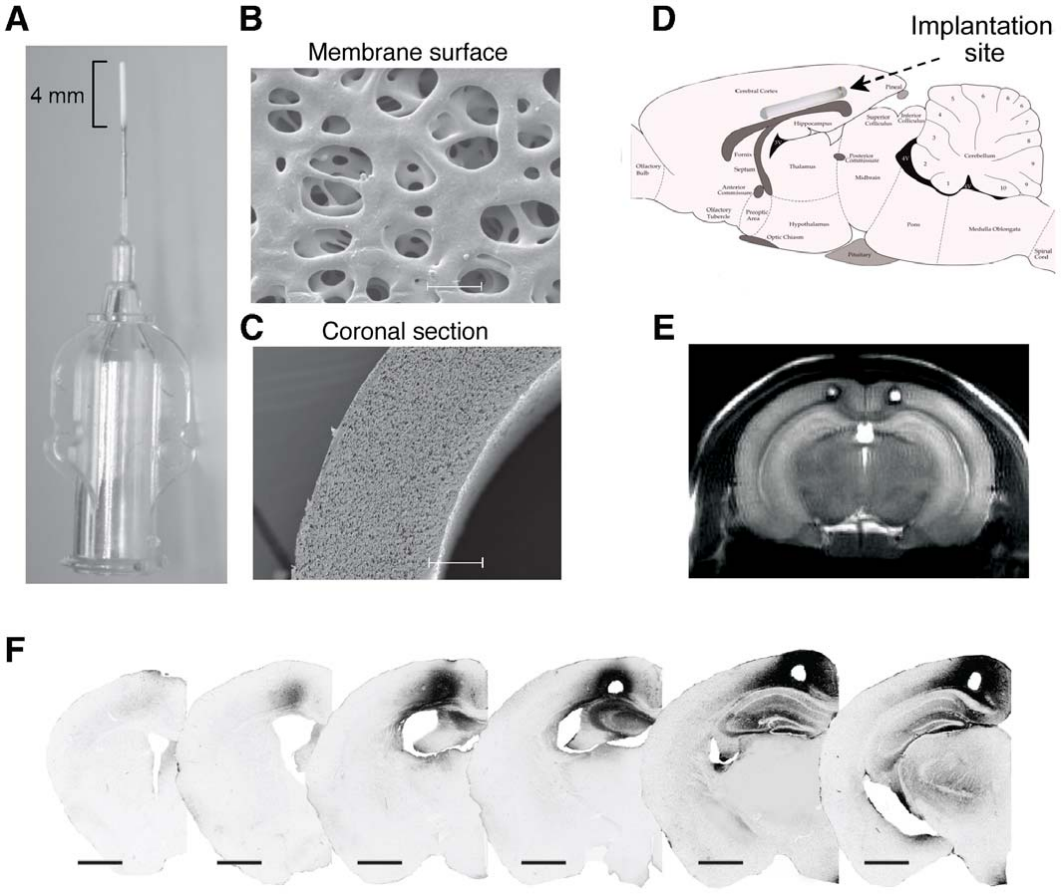
Polysulfone (PS) hollow fiber capsule use in mouse brain implantation. (**A**) Macroscopical appearance of a PS hollow fiber capsule used for the encapsulation of C2C12 cells. A 4 mm PS membrane glued and connected to a stainless steel tip facilitate the loading of cells, and the plastic hub serves to plug in a Hamilton syringe containing the cells. (**B**) The membrane surface of the PS-membrane imaged with a SEM microscope at ×10,000 reveals its porosity corresponding to a cut-off of ∼100 kDa. Scale bar 2 µm. (**C**) The coronal section of the PS-membrane imaged with a SEM microscope at ×1000 shows an even homogeneous wall structure. Scale bar 20 µm. (**D**) Sagittal schematic representation of the implantation site in the posterio-parietal cortex of a mouse brain. (**E**) MRI coronal image from an APP23 mouse brain showing the placement of the polymeric cell implants following bilateral implantation. (**F**) Immunohistochemical coronal sections of an APP23-scFvβ1 mouse brain revealing the extent of diffusion of scFvβ1 around the site of implantation. Magnification 1×, scale bar 1 mm.

Next, we designed a device for *in situ* delivery of scFvβ1 in the mouse brain. To this end, we made use of polysulfone hollow fiber membranes with an inner diameter of 280 µm, outer diameter of 360 µm and a length of 4 mm (**Figure 2A**) to be loaded with C2C12 mouse myoblast cells engineered and selected for continuous secretion of scFvβ1 antibody fragments. We used a fiber membrane with a molecular weight cut-off of ∼100 kDa, allowing the diffusion of the ∼27 kDa scFvβ1 fragments. Scanning electron microscopy was performed to assess the homogeneous surface structure and porosity of the hollow fiber membrane (**Figure 2B, C**). The hollow fiber capsules loaded with the selected C2C12-scFvβ1 cells were implanted in the posterio-parietal cortex of APP23 mice (**Figure 2D**), a mouse-model of AD-like pathology created by overexpressing human APP with the Swedish mutation [15]. Localization of the polymeric cell implants in the brain cortex of APP23-scFvβ1 and APP23-mock mice was visualized from coronal *in vivo* images using an MRI system interfaced to a 14.1 Tesla magnet (**Figure 2E**). Four weeks following the implantation, the local tissue distribution of the scFvβ1 was determined after an anti-histidine tag immunohistochemical detection in paraformaldehyde-fixed brain sections of APP23-scFvβ1 mice (**Figure 2F**). scFvβ1 staining was detected over a maximal distance of 2 mm around the implantation site.

### Encapsulation of genetically modified C2C12-scFvβ1 cells for scFvβ1 secretion

Amongst the different C2C12-scFvβ1 clones obtained after transfection, one clone was selected according to the highest scFvβ1 secretion level and cell survival inside the polymer implant. Indeed, *in vitro* evaluation of clone #24 showed that C2C12-scFvβ1 cells were secreting 31.1±0.6 ng of scFvβ1 per 50'000 cells per 24 hours (n = 4). Once encapsulated and kept *in vitro* for 3 months, polymeric cell implants were still producing 17 ng/24 hrs of scFvβ1. Cell survival within the device was investigated *in vivo* at 3 and 6 months post-implantation, showing evenly distributed C2C12 cells intermingled with the polyvinyl alcohol (PVA) matrix (**Figure S1**). The small decrease in the secretion of the capsule 6 months post-implantation may be due a small decrease of surviving cells as well as a decrease of secretion by the C2C12 cells that have differentiated in myotubes.

### scFvβ1-treated APP23 mice display reduced anxiety-like behavior in the light and dark and elevated zero maze test, and improved working memory in the Morris water maze

**Figure 3:**
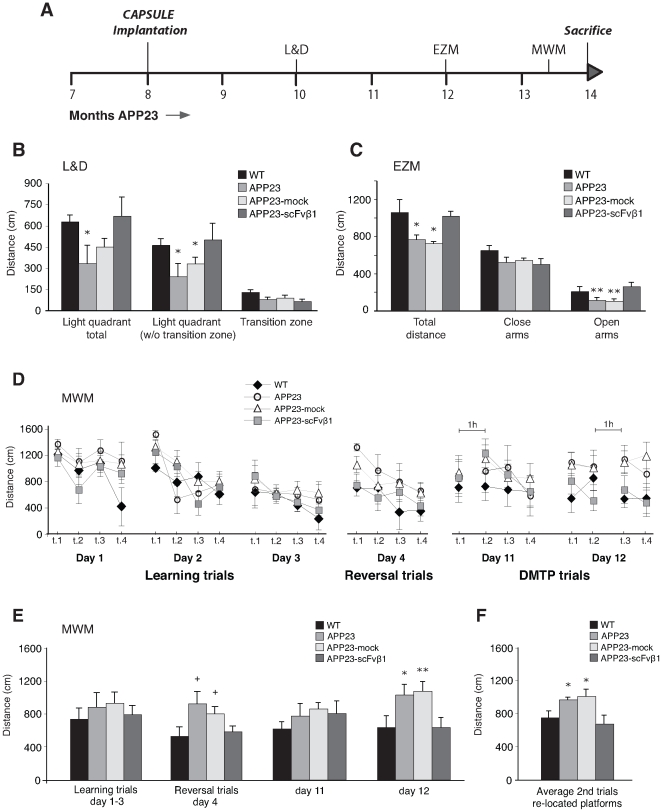
Reduced anxiety-like behavior in APP23-scFvβ1 as determined in the L&D and EZM tests, and improved working memory performance in the Morris water maze (MWM) test. (**A**) Experimental design of the behavioral battery of tests performed in APP23, APP23-mock, APP23-scFvβ1 and control aged matched WT-littermates during the course of the therapy. (**B**) The L&D test shows that WT-littermates and APP23-scFvβ1 mice display reduced anxiety-like behavior for the distance traveled in the exposed and anxiogenic open light compartment [light compartment total (F_(3,22)_ = 3.2; p = 0.042), light compartment w/o the transition zone (F_(3,22)_ = 3.4; p = 0.034), and transition zone (F_(3,22)_ = 0.67, n.s.)], when compared to APP23 and APP23 mock animals. (**C**) The EZM test show that WT and APP23-scFvβ1 mice display reduced anxiety-like behavior for their total distance traveled in the arena (F_(3,17)_ = 5.32; p = 0.009), and in the open arms (F_(3,17)_ = 5.25; p = 0.007), when compared to APP23 and APP23-mock animals. (**D, E**) The MWM test did not show significant effects during days 1-3 in the learning sessions (F_(3,24)_ = 1.42, n.s.), a tendency towards significance was observed in the reversal sessions (F_(3,24)_ = 2.38, p = 0.09). In the delayed-matching-to-place (DMTP) task APP23-scFvβ1 mice and WT-littermates displayed improved working memory on day 12 when compared to APP23 and APP23-mock, no differences were observed in the DMTP day 11 (F_(3,24)_ = 0.92, n.s.). (**F**) APP23 and APP23-mock mice showed clear impairments in their learning acquisition, as indicated by their longer distance to find the platform when the second trials for each cognitive challenge were collapsed, while APP23-scFvβ1 mice showed an improved working memory that did not differ from WT mice (F_(3,24)_ = 6.01; p = 0.003). Results are expressed in centimeters (cm) for the distance. Error bars indicate s.e.m. +p<0.1, *p<0.05 and **p<0.01 as determined by one-way ANOVA followed by the LSD post-hoc analysis.

C2C12-scFvβ1 capsules were implanted bilaterally in the posterio-parietal cortex, and animals were evaluated behaviorally (**Figure 3A**) in two different anxiety-like tests, light and dark (L&D) and elevated zero maze (EZM) (**Figure 3B, C**) and in the Morris water maze (MWM) for cognitive functions (**Figure 3D–F**).

In the L&D test, the distance traveled in the open lighted and anxiogenic compartment indicated anxiety-like behavior. Significant differences were observed between the APP23-scFvβ1 and WT-littermates groups in the total distance traveled in the light compartment compared to the APP23 group (p<0.05) (**Figure 3B**). Similarly, APP23-scFvβ1 and WT-littermates animals showed a significant difference in the distance covered in the light compartment (without the transition zone) compared to both APP23 and APP23-mock groups (p<0.05) (**Figure 3B**), indicating enhanced anxiety-like behavior in the non-scFvβ1 treated APP23 mutants. No differences between groups were found in distance moved in the transition zone, indicating a lack of changes in general exploratory behavior (**Figure 3B**).

In the EZM test, APP23 and APP23-mock mice moved less in the maze than WT mice, due to a specific reduction in both total movement in the arena (p<0.05), and in the open arms (p<0.01) (**Figure 3C**), indicating enhanced anxiety-like behavior in the non-scFvβ1 treated APP23 mutants. APP23-scFvβ1 mice differed from the untreated and mock mutants, but not from WT controls in their total movement in the arena (p<0.05), and in the open arms (p<0.001) (**Figure 3C**), indicating reduced anxiety-like behavior.

We next examined the impact of scFvβ1 treatment on cognitive functions, as evaluated 5.5 months after treatment on spatial learning and working memory functions in the MWM. No significant differences were found between WT-littermates and all APP23 groups in their distance moved to find the hidden platform over the three spatial learning sessions (days 1–3) (**Figure 3D**), and did not differ from each other in their daily average distance to reach the platform (**Figure 3E**). Following the reversal learning (day 4), the APP23-scFvβ1 and WT-littermates showed a trend to reach the platform in shorter distance compared with both control APP23 groups (p<0.1) (**Figure 3D, E**). No locomotor or visual deficits were observed between groups on the visual platform task (day 8).

Animals were then challenged to the delayed-matching-to-place (DMTP) [16] trials (days 11 and 12). On the first day no difference in performance was observed between the different groups (**Figure 3D, E**). However on the second day of the DMTP trials, APP23 and APP23-mock mice showed impaired performance with regards to both WT (p<0.05) and APP23-scFvβ1 (p<0.01) mice (**Figure 3D, E**). Therefore, scFvβ1 treatment improved mice working memory to re-locate the platform in the novel position as indicated by their shorter average escape distances (**Figure 3E**). To further assess the effectiveness of the treatment in learning acquisition, we analyzed a compound measure of mice's behavior by collapsing data corresponding to the second trials for each of the cognitive challenges given in the MWM (i.e., days 1, 4, 11 and 12). As shown in **Figure 3F**, this analysis indicated that WT and APP23-scFvβ1 mice performed better in their learning strategy than the APP23 and APP23-mock mice (p<0.05).

### scFvβ1 secretion in the brain parenchyma of APP23 mice and scFvβ1 capsule release

**Figure 4:**
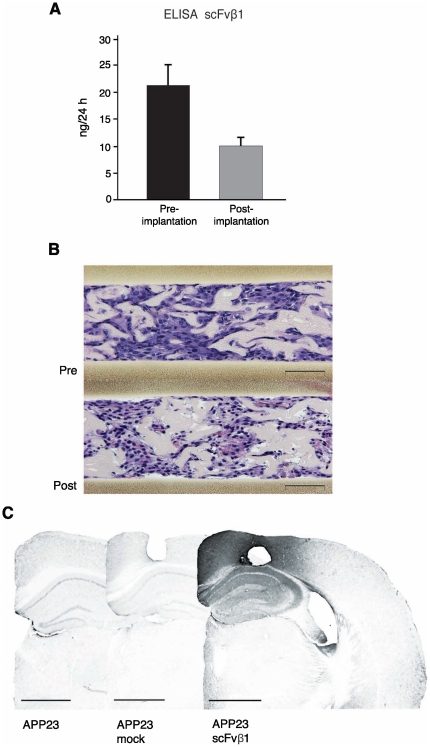
scFvβ1 release and survival of encapsulated C2C12 cells. (**A**) ELISA immunoassay reveals the amount of scFvβ1 released by C2C12-scFvβ1 polymer capsules prior to implantation, and 6 months post-explantation. (**B**) Hematoxylin–eosin staining performed on the retrieved polymeric cell implants pre- and post-implantation show the presence of numerous cells scattered within the PVA matrix. Magnification 4×, scale bar 100 µm. (**C**) Immunohistochemical detection of scFvβ1 using an anti-histidine tag antibody. Left, APP23 mouse brain without surgery. Middle, APP23 mouse that received bilateral implantation of polymer capsules with control C2C12-mock cells. Right, staining of APP23 mouse that received bilateral implantation of polymer capsules with C2C12 cells expressing the recombinant scFvβ1 antibody fragment, revealing the diffusion around the site of implantation. Magnification 1×, scale bar 1 mm.

Mice were sacrificed and the polymeric cell implants were retrieved six months post-implantation for evaluation of scFvβ1 secretion. The retrieved devices released 10±1.5 ng of scFvβ1/24 h compared to the 21±4 ng of scFvβ1/24 h at the time of implantation, confirming the sustained release of scFvβ1 fragments throughout the implantation period (**Figure 4A**). Histological analysis of the capsules confirmed the survival of the cells 6 months following implantation (**Figure 4B**). Anti-histidine tag immunohistochemical detection of scFvβ1 fragments showed the presence of the scFvβ1 surrounding the implantation site, covering the cortex (posterio-parietal region), the hippocampus (dorsal region at the CA1, CA2, CA3, CA4, and dentate gyrus) (**Figure 4C**) and, to a lesser extent, other areas near the implants and ventricles. No scFvβ1 was detected in the APP23 and APP23-mock brains (**Figure 4C**).

### Total amyloid beta is reduced after scFvβ1 delivery from encapsulated cells

**Figure 5:**
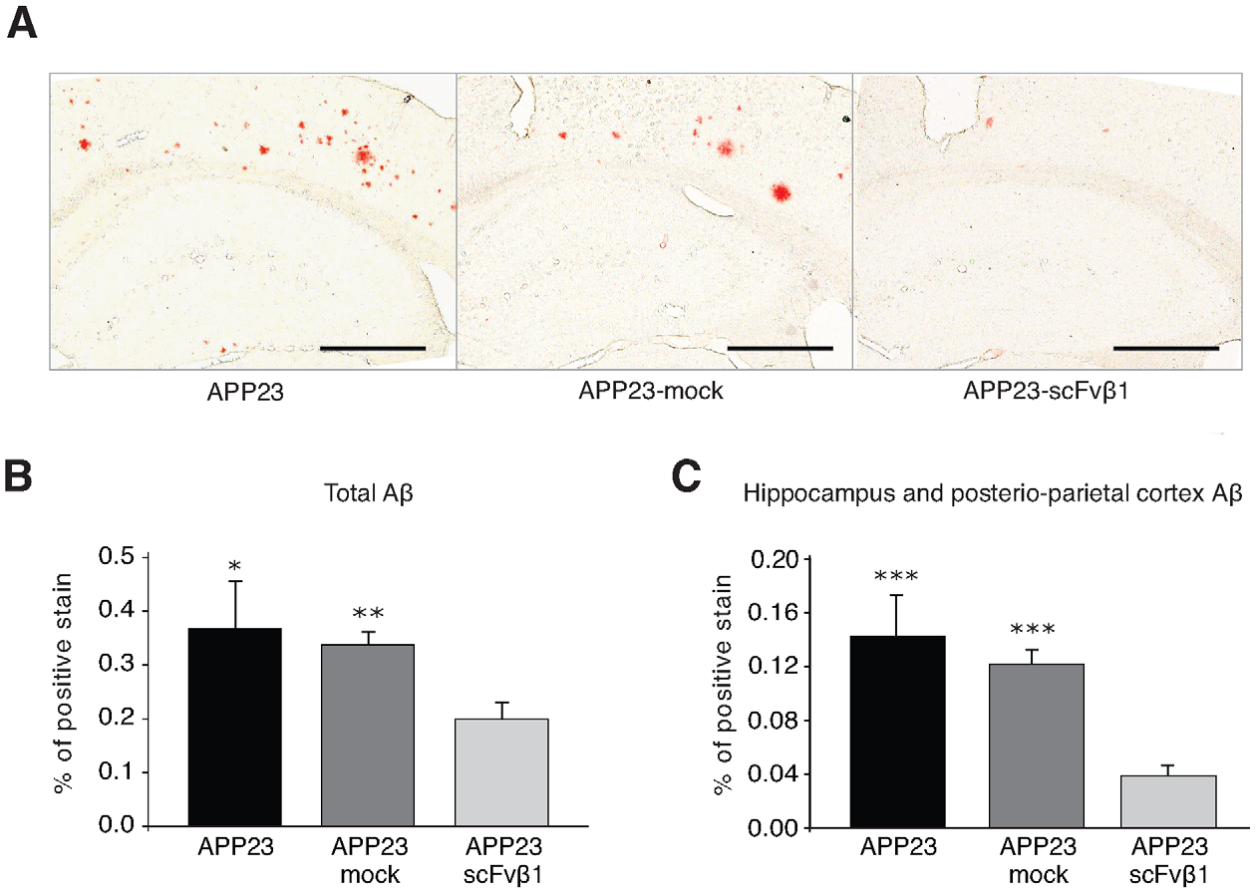
scFvβ1 decreases the accumulation Aβ *in vivo*. (**A**) Representative congophilic stained sections of Aβ plaques from brains of APP23, APP23-mock and APP23-scFvβ1 reveal the reduction of the amyloid plaque number following therapy with C2C12-scFvβ1 polymer implants. Magnification 2×, scale bar 500 µm. (**B**) Percentage of positive congophilic plaque stainings from entire brain sections is reduced in APP23-scFvβ1 mice. (**C**) Percentage of positive congophilic plaque stainings in the hippocampus and posterio-parietal cortex region is reduced on the region were the antibody diffuses from the C2C12-scFvβ1 polymer implants.

The total Aβ load was ascertained six months post-scFvβ1 treatment using immunohistochemical and congophilic stainings. In 14-month-old APP23 mice, Aβ plaque deposition was regionally distributed throughout the olfactory bulb, the cortex (although more densely concentrated in the ‘parietal’ and ‘occipital’ regions) and, to a lesser extent in the hippocampus. Congo red staining revealed that the size of Aβ insoluble plaques (**Figure 5A**) was significantly reduced in the brain slices of APP23-scFvβ1 animals with regards to both APP23 (p<0.05) and APP23-mock (p<0.01) (**Figure 5B**), and with a marked clearance in the hippocampus and posterio-parietal cortex regions as compared to APP23 and APP23-mock (p<0.001) (**Figure 5C**).

**Figure 6:**
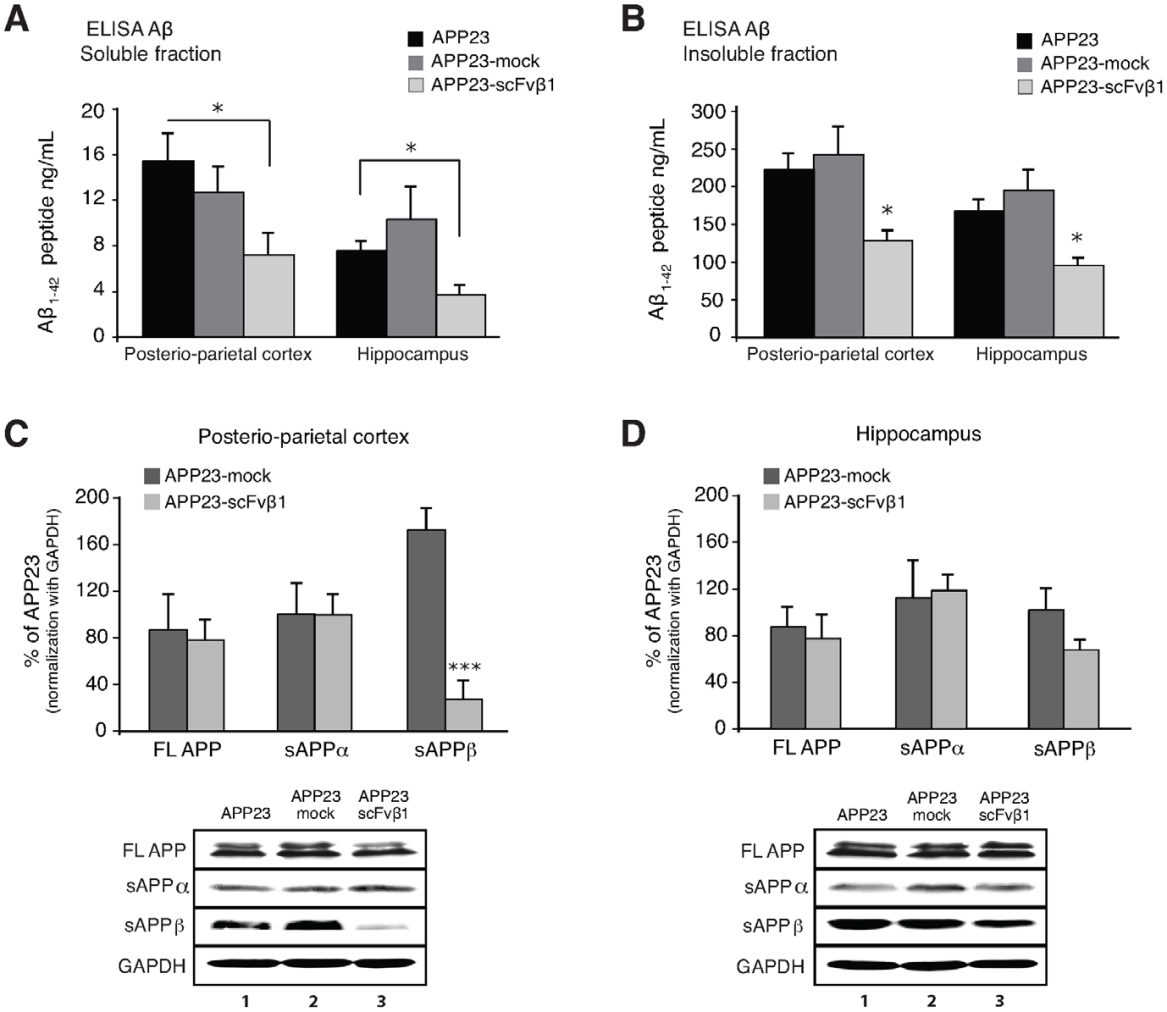
scFvβ1 decreases the production of Aβ and sAPPβ by interfering with the BACE1 cleavage of huAPP *in vivo*. (**A**, **B**) Aβ_1–42_ ELISA from brain protein extracts from soluble and insoluble fractions. (**A**) Aβ levels from the TBS soluble fractions are significantly reduced in the posterio-parietal cortex and hippocampus in APP23-scFvβ1 mice when compared to APP23 mice. (**B**) Aβ levels of the Triton-X100 insoluble fraction are significantly reduced in the posterio-parietal cortex and hippocampus region of APP23-scFvβ1 treated mice when compared to both APP23 and APP23-mock. (**C, D**) Western blot analysis measured by the protein/GAPDH ratio are analyzed and plotted as the % change against APP23 protein levels. Full length APP, and the soluble amyloid protein precursor alpha (sAPPα) display no significant changes, while soluble amyloid precursor protein beta (sAPPβ) is considerably reduced after exposure to scFvβ1 *in vivo* in the posterio-parietal cortex. Representative immunoblots are depicted below the graphs (**C, D**). Error bars indicate s.e.m. *p<0.05, **p <0.01 and ***p<0.001 as determined by one-way ANOVA followed by LSD post-hoc analysis.

ELISA from brain homogenates revealed that the soluble Aβ levels were significantly lower in APP23-scFvβ1 in the posterio-parietal cortex and hippocampus as compared to APP23 (p<0.05) (**Figure 6A**), and a trend was observed with APP23-mock (p<0.1). The insoluble levels of Aβ were significantly reduced in scFvβ1 antibody treated animals in the posterio-parietal cortex and hippocampus (p<0.05) (**Figure 6B**), confirming the observations from the congophilic quantification.

Microhemorrhages and cerebral amyloid angiopathy were scarce in all groups (data not shown). No differences between treated and non-treated animals were observed.

### The β-site cleavage of huAPP is affected in scFvβ1 treated animals

Western blot analysis from the brain homogenates showed an overall 80% reduction of the soluble APPβ in the posterio-parietal cortex of APP23-scFvβ1 animals (p<0.001) (**Figure 6C**). The β-secretase cleavage was less affected in the hippocampus accounting for only a 30% reduction of sAPPβ (p<0.2) (**Figure 6D**), suggesting that the majority of the antibody diffused in the cortical region where the capsule was implanted. The product of α-secretase cleavage sAPPα and the amount of full length human APP (FL huAPP) were not significantly affected by the expression of scFvβ1 (**Figure 6C, D**).

## Discussion

Currently one of the principal challenges for the clinical use of antibody fragments is to modify the pharmacokinetic properties to achieve a balance of prolonged retention at the target site and fast systemic clearance. Modifications of scFv's such as PEGylation are able to increase the half-lives up to 14-fold *in vivo* [17], but significantly lower tissue penetration. Other explored routes such as nasal administration of scFv's are able to increase their availability inside the brain [18]. However, the approaches require continuous interventions to achieve therapeutic effects [9], [10]. Here, we have show the feasibility of an original way to continuously release scFv antibody fragments *in situ* to enhance the availability and accumulation to the target tissue.

The use of single chain antibodies (scFv) for passive immunization constitutes an alternative strategy to immunotherapies based on monoclonal antibodies [9], [19], [20], [21]. The smaller size of these molecules (approximately 27 kDa) improves tissue penetration [22], while the absence of the Fc portion may enhance the blood-brain barrier infiltration and preclude activation of the complement system and inflammatory reactions. Limitations for their therapeutic use for systemic delivery are related to their shorter half-life in the blood (2 h) compared to full antibodies (1–2 w) [1], [22]. The local delivery of antibody fragments presents a clear advantage over applications when systemic delivery is not safe, or not efficient due to poor bioavailability.

As shown in the *in vitro* studies, immunotherapy with scFvβ1 could not only prevent deposition and oligomerization of Aβ by binding to the soluble form but also reduce its production by binding membrane-exposed huAPP due to its increased capacity to access the epitope. *In vivo* sustained release of scFvβ1 fragments from the polymeric cell implants considerably reduced the Aβ_1–42_ burden in the brain of APP23 mice. Results indicated that clearance of Aβ by scFvβ1 most likely implicates the interaction of the antibody with monomeric and small oligomeric Aβ assemblies, either by forming a soluble-complex scFvβ1/Aβ that impedes accumulation, and/or as a membrane-complex huAPP/scFvβ1 that blocks the production of Aβ. Presently, we cannot exclude that alone the overexpression of human APP with the Swedish mutation may account for the behavioral phenotype observed in the APP23 mice, and that scFvβ1 treatment neutralizes the potential pathogenic effect of human mutant APP rather than of Aβ accumulating in the brain. On the other hand, *in situ* production of scFvβ1 was found to affect primarily the generation of Aβ by β-secretase as well as to interact with extracellular Aβ, where sAPPα and full-length APP were unchanged suggesting an Aβ-driven process. In addition, we discard that reduction of Aβ load occurs via microglial phagocytosis [19], [21], given that the scFvβ1 fragment lacks the Fc-region of the parental β1 antibody we argue in favor of a preventive mode of action where scFvβ1 inhibits the initial accumulation of Aβ species.

Behavioral evaluation during the course of the immunotherapy showed that reduction of Aβ levels after scFvβ1 delivery modified behavioral traits related to anxiety and working memory in the APP23 mice. Aβ progressive accumulation has been previously reported to increase anxiety in numerous AD mouse models [23], [24] and to deteriorate cognitive-associated areas related to spatial and working memory [25], [26] and these findings were confirmed in our study. They also showed improved learning strategies during the second MWM learning trials, and displayed improved working memory in the DMTP paradigm.

CAA frequency and severity was not affected by immunization. Because robust CAA and microhemorrhages have only been reported in >19 month-old APP23 mice [13], [25], [27], it is conceivable that the lack of differences between the various groups of animals is caused by the low presence of CAA at the time of sacrifice [25], [27].

In summary, we have established a novel *in situ* passive immunotherapy strategy using an immunoisolated allogeneic implant capable of expressing, processing, and secreting scFv antibodies against the Aβ peptide in the brain parenchyma of APP23 AD transgenic mouse. We show that local and sustained release of scFvβ1 fragments from polymeric cell implants over a 6-month treatment significantly reduce the Aβ_1–42_ burden, and modify behavioral traits related to anxiety and working memory.

The present data demonstrates the proof of principle of an innovative technology for the sustained release of antibodies *in vivo*. The use of polymeric cell implants is a promising alternative tool to the current passive vaccination strategies. The potential therapeutic advantages of this singular immunoisolated device rely on its capacity to release for long-term antibodies and/or antibody fragments, while its design allows retrieveabiity for either replacement or interruption of the treatment [28], [29], [30]. This type of technology has the possibility to be scaled-up by using enlarged cell encapsulation devices to potentially achieve physiological levels of antibodies for local and systemic applications. Indeed the technique has been previously validated for the systemic delivery of erythropoietin in mice [31], and for the local brain delivery of neurotrophic factors in rodents [30], non-human primates [32], and in two clinical trials [28], [33]. Beyond its potential for the treatment of AD, this technology could also benefit other major diseases presently treated by antibody administration.

## Materials and Methods

### *In vitro* test of β1 and scFvβ1 antibodies

CHO cells ectopically expressing the Swedish variant of human APP were incubated in the presence of 1.88 µM of antigen-binding site (scFvβ1 or β1) [11], [13], or in the presence of 500 nM DAPT added to the cell culture media. Cells were metabolically labeled for 3 h with ^35^S-methionine and -cysteine (0.05 mCi/dish; Perkin Elmer). The labeled APP and APP fragments were immunoisolated from cell culture media or from cell lysates with appropriate antibodies and separated electrophoretically as previously described [11].

### Generation of a stable C2C12 cell line for encapsulation

C2C12 mouse myoblasts (ECACC) at 80% confluency were transfected using Lipofectamine 2000 (Invitrogen) with 3 µg of a ScaI-linearized pRK5 plasmid for expression of scFvβ1-His_6[11]_, as control we used cells transfected with the pRK5 plasmid without the transgene (mock). We used 1.5 µg of a *Pvu*I-linearized pCDNA3 plasmid for geneticin antibiotic selection of transfected cells. Three positive stable clones that maintained high level of expression of scFvβ1 for several months in culture were selected for their encapsulation into hollow fiber membranes.

### Hollow fiber capsule design

Stainless steel tips (EFD) (OD: 230 µm; ID: 100 µm) were detached from their hub and connected to the tip of 4 mm long polysulfone (PS) hollow fiber semipermeable membranes (Minntec) (OD: 360 µm; ID: 280 µm; molecular weight cutoff: 100 kDa) using a photo-polymerized acrylic-based glue (Ablestic Laboratories). Hollow fiber membranes were filled with a polyvinyl alcohol (PVA) sponge (Rippey Corporation) used for cell anchorage and were obtained using a hollow drill with an internal diameter corresponding to the inner dimensions of the capsule. The PVA rods were sonicated in ultra pure water and dried. The matrices were inserted into the 4 mm long semi-permeable PS hollow fibers and sealed. Capsules were sterilized with ethylene oxide and kept 10 days at room temperature to eliminate traces of gas.

### SEM imaging

PS hollow fiber membranes structure was visualized using a Philips XLF30 field emission gun scanning electron microscope (FEG SEM), equipped with an Everhart-Thornley secondary-electron (SE). Membranes were dehydrated in alcohol baths from 70 to 100% followed by a 400 Å gold plasma coating for visualization at different resolutions.

### Cell loading

C2C12-scFvβ1 clones (#9, 15, and 24) were harvested using 0.125% trypsin-EDTA and diluted with 50% DMEM 5% FBS +50% HBSS to achieve a suspension of 50,000 cells/µl culture medium. Using a 50-µl syringe (Hamilton) fitted with an adaptor hub, 1 µl of cell suspension was injected into the capsule. The hubs and steel tip were removed and the extremity of the capsules sealed. The capsules were washed in HBSS 1% FBS for 1 hour and then transferred to DMEM, 10% FBS (5% CO_2_, 37°C) for 21 days before implantation into the brain cortex of mice.

### scFvβ1 detection

PVC plates were coated with Aβ_1–40_ (10 ng/µL). scFvβ1 standards were concentrated from culture medium using his-tag columns (GE healthcare) and purified using fast protein liquid chromatography. As primary antibody we used culture media supernatant of capsules expressing the scFvβ1. As a secondary antibody we used anti-his tag biotinilated (Serotec) and the substrate solutions according to the manufacturer (R&D systems). To detect the presence of metabolically active C2C12-mock cells inside capsules, a lactate assay kit was used (BioVision) (data not shown).

### Animal Care and Treatment

Ethic statements: All animal experiments were approved by the SCAV (Service de la consommation et des affaires veterinaires) in the Canton de Vaud, and carried out in accordance with the European Community Council Directive (86/609/EEC) for care and use of laboratory animals. Permit number: 1935.

Subjects were heterozygous females APP23 mice and their non-transgenic WT-littermates generated as previously described [15]. These mice express human APP751 cDNA with the Swedish double mutation under control of the neuron-specific mouse Thy-1 promoter fragment. They produce a seven-fold excess of huAPP compared to the endogenous murine APP.

Eight-month age matched female APP23 mice were housed in 12 h light/dark cycle, with *ad libitum* access to food and water. Deeply anesthetized animals were placed into the stereotaxic frame (Kopf Instruments) equipped with a precise micromanipulator with a horizontal arm in a 79° angle. Four-millimeter long capsules were bilaterally implanted in the cortex (anterior-posterior: −1.1 mm, lateral: ±1.2 mm, ventral: −5.5 mm, tooth bar: −7 mm, according to the atlas of Paxinos and Franklin [34]) of 16 female APP23 mice. The first group consisted in 9 APP23 mice implanted with C2C12-mock, and the second with 7 APP23 mice implanted with C2C12-scFvβ1 capsules.

### Capsule histology

Following the ELISA-scFvβ1 immunoassay, capsules retrieved from the animals were fixed overnight in 10% formalin and 1% picric acid and dehydrated under an alcohol cycle in preparation for glycol–methacrylate embedding (Leica Instruments). The capsules were cut at 9 µm-thickness using a LEICA microtome equipped with glass knifes and stained with hematoxylin–eosin (HE).

### Behavioral analysis

Behavioral testing was performed in female APP23 mice (n = 21) and their WT-littermates (n = 7) during the light cycle period (8 am to 2 pm). In all tests, mice trajectories were recorded with a vertically mounted camera and analyzed with a video tracking software (Ethovision 3.1.16, Noldus). In order to maximize homogeneity of groups before scFvβ1 capsule implantation, 7 month-old APP23 mice (*n* = 21) were tested for anxiety-like, locomotor and exploration behaviors in the elevated plus maze (EPM), the open field and the novel object (OF/NO) reactivity test (**Methods S1**) and subsequently we matched them so that not significant differences were observed between the subgroups (APP23 *n* = 5, APP23-mock *n* = 9, APP23-scFvβ1 *n* = 7) (**Table S1**).

To evaluate the behavioral impact of the scFvβ1 treatment, three tests were administered at different time points after capsule implantation (**Figure 3A**). (a) *Light Dark test* (L&D) (**Methods S1**); (b) *Elevated zero maze test* (EZM) (**Methods S1**); and (c) *Morris water maze* (MWM) (**Methods S1**).

### Magnetic resonance imaging (MRI)

Five APP23-mock, and 5 APP23-scFvβ1 mice were anesthetized using 1.3±0.2% of isoflurane in oxygen using a nose mask. Body temperature was kept at 37±0.5°C. Images were acquired on an MRI System (Varian) interfaced to a 14.1 Tesla magnet with a 26-cm horizontal bore (Magnex Scientific). A home-built quadrature surface coil consisting of two geometrically decoupled 14-mm-diameter single loops was used as a transceiver. Localizer images were obtained in the coronal plane using a multislice fast spin echo protocol with an echo time of 60 ms, a repetition times of 5000 ms, a slice thickness of 0.6 mm and an isotropic in-plane resolution of 78 µm.

### Analysis of brain samples

Mice were deeply anesthetized by an overdose of pentobarbital and transcardially perfused with ice-cold PBS. The brain was recovered and capsules were removed and placed in DMEM 10% FBS at 37°C, 5% CO_2_. Brains were sagittally sectioned in two; the hippocampus and cortex of the left hemisphere were immediately dissected for protein extraction, and the right hemisphere was immediately fixed in 4% paraformaldehyde (Fluka-Sigma) for 2 hours and then transferred into 25% sucrose in PBS and placed at 4°C overnight. Twenty-five µm thick coronal sections were harvested on a freezing stage sliding microtome (Leica SM2400).

### Immunohistochemistry

For immunofluorescence studies, slices were tested with the following primary antibodies: anti-amyloid beta 4G8 (Acris), mouse anti-glial fibrillary acidic protein (Novus Biologicals), and anti-Iba1 (Osaka, Japan). As secondary antibodies we used 488- and/or 568-Alexa fluor dyes (Molecular Probes). For detection of the scFvβ1, an anti-histidine tag mouse monoclonal antibody (Serotec) was used, followed by peroxidase treatment M.O.M (Vector Laboratories), and revealed with the 3,3′-diaminobenzidine (DAB) (Pierce). We performed congo red histology as previously described [35], [36] on ten coronal brain sections (100 µm apart; every 4^th^ sections) taken from each animal in the region where the capsule was placed. Entire brain slices were captured in the bright field with a motorized stage on the Leica DM5500 microscope (software: Leica LAS) at a 10× resolution. Each brain slice was segmented from the background to obtain the brain surface followed by the quantification of the size of the amyloid plaques. Both processes were performed through different channel manipulation of the RGB images and then by object detection. Artifacts were avoided by filtration on shape and size. The semi-automated journals were performed with METAMORPH 7.5 (Universal-Imaging). Cerebral amyloid angiopathy and microhemorrhages were quantified using a double staining with 4G8 antibody in DAB (described above), and counterstained with the Prussian blue method for hemosiderin-positive microglial cells in eight coronal brain sections (150 µm apart) throughout the sector where the capsule were implanted.

### Protein extraction analysis

We performed soluble (Tris-buffered saline (TBS)), detergent-soluble (TBS with 1% Triton X-100) and insoluble (5 M GuHCl) extracts as previously described [37]. Fractions were analyzed for the quantification of human Aβ_1–42_ using a colorimetric sandwich ELISA kit (Biosource). Western blot analysis from protein extracts were separated using sodium dodecyl sulfate polyacrylamide elecrophoresis and transferred onto nitrocellulose membranes, where they were probed with the primary mouse monoclonal CT55 (Sigma) antibody against the C-terminal APP; the mouse anti-Aβ 6E10 (Signet) for sAPPα; and the mouse monoclonal 6A1 (IBL) for sAPPβ-Swedish. Primary antibodies were detected with Alexa Fluor 680 anti-goat or anti-rabbit antibodies (Invitrogen-Molecular Probes) using an Odyssey Infrared Imaging System (LI-COR Biosciences). Densitometry analysis was performed using the Odyssey application software (Version 2.1) (LI-COR Biosciences) and normalized with the housekeeping protein glyceraldehyde 3-phosphate dehydrogenase (GAPDH) (Abcam) using an anti-mouse Alexa Fluor 800 antibody.

### Statistics

Pre-implantation and post-implantation behavioral data were analyzed using a one-way analysis of variance (ANOVA) followed by an LSD post-hoc test, where appropriate. Water maze data were analyzed using ANOVA for repeated measures for general performance across spatial learning in trials; one-way ANOVAs were applied on block data for each testing day followed by a post-hoc LSD test. Significance of results was accepted at p≤0.05. Data are expressed as means ± S.E.M.

## Supporting Information

Figure S1 **Hematoxilin-eosin (HE) staining performed on retrieved capsules recovered from** ***in vivo*** **intracranial implantations in C57BL/6 mice.** (**A**, **B**) HE staining of capsules retrieved 3 months post-implantation showing the presence of numerous scattered cells within the PVA matrix. (**C**, **D**) HE staining of capsules retrieved 6 months post-implantation showing the presence of numerous scattered cells within the PVA matrix. Magnification 10×, scale bar 100 µm.
(TIF)

Table S1 **Distribution of three different groups of APP23 mice following behavioral screenings.** Seven month-old female APP23 mice (n = 21) were subjected to behavioral tests in the elevated plus maze test, the open field test and the novel object test before capsule implantation. Mice were matched and homogeneously distributed according to their body weight, behavioral traits of anxiety, locomotion and exploration. Analysis of variance confirmed that significant differences did not exist between the subgroups (APP23, APP23-mock & APP23-scFvβ1). Results were analyzed using a one-way analysis of variance (ANOVA), significance of results was accepted at p≤0.05.
(DOCX)

Methods S1 **Behavioral tests.**
(DOCX)
