# Supplementary figures and images for: Chronic Delivery of Antibody Fragments Using Immunoisolated Cell Implants as a Passive Vaccination Tool
Source: PLoS One. 2011 Apr 20;6(4):e18268. doi: 10.1371/journal.pone.0018268 (PMC3080361; doi:10.1371/journal.pone.0018268)

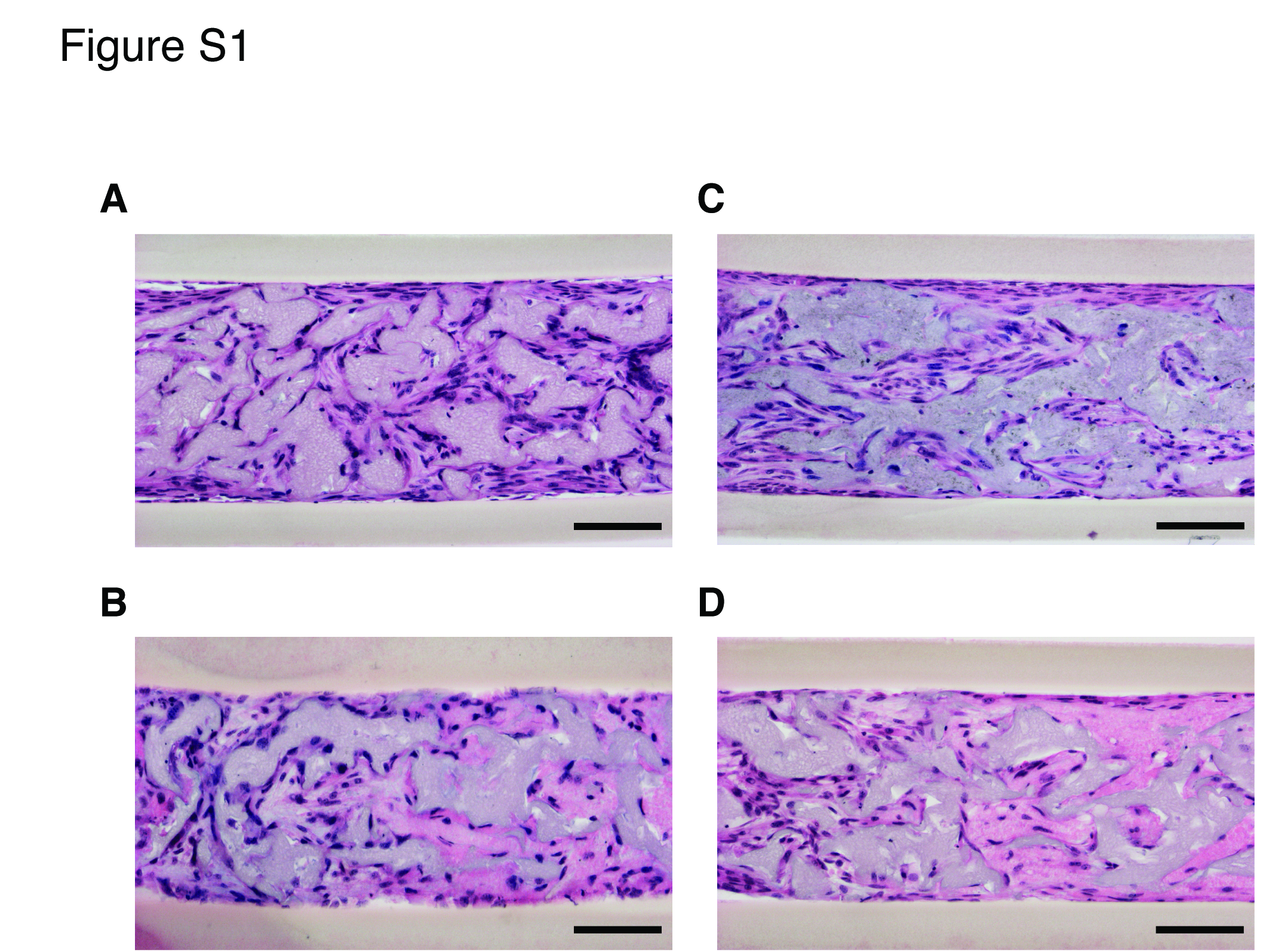

Supplement: Figure S1 — Hematoxilin-eosin (HE) staining performed on retrieved capsules recovered from in vivo intracranial implantations in C57BL/6 mice. (A, B) HE staining of capsules retrieved 3 months post-implantation showing the presence of numerous scattered cells within the PVA matrix. (C, D) HE staining of capsules retrieved 6 months post-implantation showing the presence of numerous scattered cells within the PVA matrix. Magnification 10×, scale bar 100 µm. (TIF) [file pone.0018268.s001.tif]
