# Supplementary material for: Chronic Delivery of Antibody Fragments Using Immunoisolated Cell Implants as a Passive Vaccination Tool
Source: PLoS One. 2011 Apr 20;6(4):e18268. doi: 10.1371/journal.pone.0018268 (PMC3080361; doi:10.1371/journal.pone.0018268)
Supplement: Table S1 — Distribution of three different groups of APP23 mice following behavioral screenings. Seven month-old female APP23 mice (n = 21) were subjected to behavioral tests in the elevated plus maze test, the open field test and the novel object test before capsule implantation. Mice were matched and homogeneously distributed according to their body weight, behavioral traits of anxiety, locomotion and exploration. Analysis of variance confirmed that significant differences did not exist between the subgroups (APP23, APP23-mock & APP23-scFvβ1). Results were analyzed using a one-way analysis of variance (ANOVA), significance of results was accepted at p≤0.05. (DOCX) [file pone.0018268.s002.docx]

**Table S1**

| **EPM**  **(elevated plus maze)** | **F _(2,11)_** | **p** |
| --- | --- | --- |
| Total distance (cm) | 3.22 | 0.08 |
| Distance open arms (cm) | 2.05 | 0.18 |
| Frequency entries arms | 0.94 | 0.42 |
| Latency open arms | 1.93 | 0.19 |
| Percent time center | 0.07 | 0.93 |
| Percent time close arms | 0.20 | 0.82 |
| Percent time open arms | 0.49 | 0.63 |

| **OF**  **(open field)** | **F _(2,18)_** | **p** |
| --- | --- | --- |
| Total distance (cm) | 0.65 | 0.54 |
| Frequency entries center | 2.10 | 0.15 |
| Distance in the exterior (cm) | 0.39 | 0.68 |
| Percent time center | 0.85 | 0.44 |
| Percent time interior | 1.94 | 0.17 |
| Percent time exterior | 1.70 | 0.21 |
| velocity (cm/s) | 0.50 | 0.61 |

| **NO**  **(novel object)** | **F _(2,18)_** | **p** |
| --- | --- | --- |
| Total distance (cm) | 0.49 | 0.62 |
| Distance center (cm) | 1.19 | 0.33 |
| Distance wall (cm) | 0.36 | 0.70 |
| Frequency visits object | 0.77 | 0.48 |
| Latency to investigate the object | 0.56 | 0.58 |
| Percent time touching the object | 0.82 | 0.46 |
| Velocity (cm/s) | 0.22 | 0.81 |
