# Supplementary material for: Chronic Delivery of Antibody Fragments Using Immunoisolated Cell Implants as a Passive Vaccination Tool
Source: PLoS One. 2011 Apr 20;6(4):e18268. doi: 10.1371/journal.pone.0018268 (PMC3080361; doi:10.1371/journal.pone.0018268)
Supplement: Methods S1 — Behavioral tests. (DOCX) [file pone.0018268.s003.docx]

**METHODS S1**

**Behavioral tests**.

Pre-implantation:

*(1) Elevated plus maze* (EPM): this apparatus consists of two opposite open arms and two opposite closed arms (30×5×14 cm) arranged at right angles. The arms extended from a common central platform (5×5 cm) that gave access to all arms. The maze was elevated 70 cm above the floor under dim and dispersed light conditions (50 lux). Mice were placed on the central platform facing a closed arm and allowed to explore the maze for 5 min. The total distance moved, time spent in the center, open and closed arms, number and latency of entries to the open and closed arms were analyzed. Mice that fell down from the arena were removed from the statistical analysis.

*(2) Open field/novel object* (OF/NO) test was conducted under dim and dispersed light conditions in a white quadratic box (100×100×37 cm), divided into four square compartments (50×50×37 cm), allowing the evaluation of locomotor and exploratory activity of up to four mice at the same time. Each mouse was placed into the center of the arena and allowed to move freely for 11 min. The total distance moved, frequency of entries to the center, time and percent time in the center of the OF were analyzed. Exploratory behavior was assessed using a novel object (NO) test. The NO was performed immediately after the OF test. A small, metallic (non-reflective) object (3 × 1.5 × 5 cm) was placed into the center of the open field while the mouse was inside. Mice were given then 5 min to freely explore the novel object. The time spent in the center and the periphery of the compartment, number and latency of entries to the center, total distance moved in the center were analyzed.

Post-implantation:

*(1) Light Dark test (L&D)*: this apparatus consists of two interconnected compartments (50x50x37): one (‘safe’) dark and one (‘aversive’) illuminated. The test measures anxiety-like behaviors by confronting mice with a conflict between their innate aversion to brightly illuminated areas and their spontaneous exploratory activity. Each mouse was placed in the center of the arena and allowed to move freely for 5 min. A virtual ‘transition’ zone (7 cm^2^) located in the light compartment just outside the entry to the dark quadrant was considered for analysis of exploratory behavior. Mice that remained immobile in the arena were removed from the statistical analysis. The total distance moved and the time spent in each of the zones were measured, with movement and time spent in the light compartment used as indexes of anxiety-like behavior.

*(2) Elevated zero maze test (EZM)*: the apparatus consists of an elevated annular platform (outer diameter 46 cm, width 5.5 cm) with two opposite, enclosed quadrants and two open quadrants. Anxiety-like behaviors are reflected by avoiding the open, potentially threatening, and open arms. Each session lasted 5 minutes and started by placing the mouse in the entry of one closed sector. The total distance moved and time spent in both the open and the closed arms were analyzed, with low levels in the open arms reflecting anxiety-like behaviors. Mice that fell down from the arena were removed from the statistical analysis.

*(3) Morris water maze (MWM) test*: a 140 cm diameter pool containing a hidden escape platform submerged 1 mm from the surface of the water was used. Following a habituation session (day 0), mice were submitted to different protocols to sequentially assess their spatial learning abilities (days 1-3), reversal learning (day 4), visual acuity (day 8, visual platform test), and working memory capabilities [days 11-12, delayed matching-to-place (DMTP) paradigm]. Each session consisted of four trials (inter-trial intervals 15 min). Each trial started with the mouse facing the wall randomly at one of four possible positions. If the mouse did not find the platform within 60 s, it was guided towards it. Each mouse remained on the platform for 15 s before being taken out and placed in a holding cage warmed by a red heating bulb. On day 0, mice were given a free swim trial in the pool (without platform) for 2 min. At the end of this trial, the platform was rapidly placed in the pool and the mouse allowed to stay on it for 15 s. Spatial learning sessions were conducted on three consecutive days (days 1–3). On day-4, the platform position was changed to the opposite quadrant (SE). On day-8, mice were given two trials with a cued platform. On days 11 and 12, mice were submitted to the DMTP protocol that consisted of 4 daily trials and the hidden platform hidden in a new location each day, with the intertrial interval (ITI) between trials 1 and 2 ranging for 1 hour for day 11, and ITI between trials 2 and 3 ranging for 1 hour for day 12.
